# A microfabricated nerve-on-a-chip platform for rapid assessment of neural conduction in explanted peripheral nerve fibers

**DOI:** 10.1038/s41467-018-06895-7

**Published:** 2018-10-23

**Authors:** Sandra Gribi, Sophie du Bois de Dunilac, Diego Ghezzi, Stéphanie P. Lacour

**Affiliations:** 1 Bertarelli Foundation Chair in Neuroprosthetic Technology, Laboratory for Soft Bioelectronics Interface, Institute of Microengineering, Institute of Bioengineering, Centre for Neuroprosthetics, Ecole Polytechnique Fédérale de Lausanne (EPFL), 1202 Geneva, Switzerland; 2 Medtronic Chair in Neuroengineering, Center for Neuroprosthetics and Institute of Bioengineering, School of Engineering, École Polytechnique Fédérale de Lausanne (EPFL), 1202 Geneva, Switzerland

## Abstract

Peripheral nerves are anisotropic and heterogeneous neural tissues. Their complex physiology restricts realistic in vitro models, and high resolution and selective probing of axonal activity. Here, we present a nerve-on-a-chip platform that enables rapid extracellular recording and axonal tracking of action potentials collected from tens of myelinated fibers. The platform consists of microfabricated stimulation and recording microchannel electrode arrays. First, we identify conduction velocities of action potentials traveling through the microchannel and propose a robust data-sorting algorithm using velocity selective recording. We optimize channel geometry and electrode spacing to enhance the algorithm reliability. Second, we demonstrate selective heat-induced neuro-inhibition of peripheral nerve activity upon local illumination of a conjugated polymer (P3HT) blended with a fullerene derivative (PCBM) coated on the floor of the microchannel. We demonstrate the nerve-on-a-chip platform is a versatile tool to optimize the design of implantable peripheral nerve interfaces and test selective neuromodulation techniques ex vivo.

## Abstract (web summary)

Peripheral nerves have a complex physiology and it is therefore difficult to measure axonal activity in vitro. Here the authors make a nerve-on-a-chip platform to align peripheral nerves and permit measurement of conduction amplitude and velocity along several axons in a single experiment.

## Introduction

Since the first recording of propagating intracellular action potential along a nerve fiber by Hodgkin and Huxley^1^, electrophysiology has become a leading technique to study and control properties and functions of neurons, both in vitro^1–4^ and in vivo^5–12^.

Assessing peripheral nerve function is challenging given the anisotropic nature of peripheral nerves. They spread throughout the body and vary significantly in diameter from sub-millimeter up to centimeter. Within each nerve, there are hundreds to thousands of axons, which vary in diameter (1–20 µm), degree of myelination, velocity of signal propagation (0.1–120 ms^–1^), and direction of propagation (afferent vs efferent fibers). In myelinated fibers, these signals are concentrated at the nodes of Ranvier and their electrical potential dissipates in the low-resistance, extracellular space. In addition, the morphology and the phenotype of the nerve fibers influence their response to physical or biochemical perturbation. In vivo, electrodes are implanted either around or into the nerve to record extracellular signals. The ability to differentiate fibers and measure potential disruption in signal conduction is restricted by the necessary trade-off between implant invasiveness and selectivity^13^. Consequently, monitoring neural activity in a healthy peripheral nerve or following trauma, disease, chronic conditions, or drug exposure is an intricate mission.

Recent nerve-on-a-chip models have been developed to break down the complexity found in vivo, using in vitro neuronal cultures^14–19^ or ex vivo explanted nerves^20–27^. In vitro extracellular recording interfaces are manufactured using microfabrication to ensure repeatability and enable statistically relevant sample sizes^14–19^. They consist of planar microelectrode arrays (MEAs)^14–16^ or microchannel electrodes^26,28–31^ that combine axonal guidance with high signal-to-noise ratio (SNR) recordings. High-density complementary metal–oxide–semiconductor MEA combined with built-in microfluidic channel can help detecting complex signals along individual axons, at subcellular resolution^16,32^. In vitro neural cultures reach maturation after several weeks and support homogeneous population of neurons. In two-dimensional (2D) and three-dimensional (3D) culture systems, axons usually grow up to a few millimeters length^14–19^. Although myelination can be induced under specific, appropriately timed culture conditions^14,18,19^, standard culture techniques produce unmyelinated, thin ( < 3 µm diameter) axons, with resulting conduction velocities below 2 ms^–1^^15–17^. Seeding neurons in 3D scaffolds can lead to the formation of aligned fibers mimicking nerve structure^17,19^. In this configuration, neural activity, usually compound action potentials (CAPs), is visualized using Ca^2+^ imaging^18,19^ or acquired with electrodes positioned by hand with micromanipulators^17^. Recording of single-fiber action potentials (SFAPs) remains to be achieved.

Ex vivo nerve models enable probing of explanted nerves carrying cm-long myelinated fibers. Stimulation and recording from explanted tissue are usually performed in custom-made electrode set-ups^20–27^ involving micromanipulators, hook electrodes, insulating oil baths^25^, or cuff-electrodes^24,28^. Alternatively, penetrating or suction electrodes may be used^27,28^. The resulting SNR typically allow the detection of multiple SFPA composing CAP^21^ and conduction velocity computation but these experimental techniques are cumbersome and time consuming.

Although each of the aforementioned approaches has its merits, none enables systematic monitoring and quantification of neural activity from heterogeneous ensembles of nerve fibers, reflecting in vivo anatomy and transduction. Here, we introduce an ex vivo platform that integrates a realistic 3D nerve model with precise stimulation and high-resolution recordings of neural signals (SFAP, multi-unit action potentials (MUAPs) and CAP) and computation of conduction velocity. The nerve-on-a-chip platform hosts microfabricated microchannel electrodes on glass wafer allowing for precise and reproducible layout of the microelectrodes, and rapid and consistent positioning of explanted nerve root threads through the micro-conduits, which enhances the recordings throughput from excised tissue. The electrode design enables high SNR extracellular recordings with controlled spatial and temporal registration leading to measures of neural signal amplitude, density, and velocity.

We exploit the nerve-on-a-chip platform as an efficient design tool for neuroprosthetic research focusing on implants for nerve regeneration and peripheral nerve cuffs. Regenerative microchannel implants offer a fascicular-like design with tens of parallel micro-conduits that support peripheral nerve regeneration and embed microelectrodes that communicate with the regenerated axons^10,33–36^, whereas the microchannel design amplifies the extracellular neural signal amplitude^26,28^. SFAP are recordable in microchannel as short as 4 mm^34^, whereas nerve fibers can regenerate in vivo up to 6 mm through bundle of 100 × 100 µm^2^ cross-section microchannels^33^. Such implants are useful tools to both understand nerve regeneration and design bidirectional interfaces for artificial limb control, yet current designs usually contain only one electrode site per microchannel, limiting recording capability. Although the fabrication and interfacing of high-density electrode arrays within microchannel implants remain a challenge, we can optimize the design of microchannel electrodes in vitro. In a first study, we use the nerve-on-a-chip platform to test microchannel length, electrodes position and number, and neural signal waveform against the ability to compute conduction velocity using a velocity selective recording (VSR) algorithm.

In a second study, we employ the platform to test a nerve conduction block strategy based on thin coating of optoelectronic organic semiconductor films. Conditions including amputation^37,38^, spinal cord injury^10^, or retinal degeneration^39^ call for efficient nerve conduction blocking to reduce pain or silence neural hyperactivity. Feyen et al.^4^ introduced controlled silencing of neuronal activity through opto-thermal transduction in explanted and cultured neuronal network using a semiconductive poly(3-hexylthiophene):phenyl-C61-butyric acid methyl ester (P3HT:PCBM) blend polymer. This blend is compatible with thin-film technology and microfabrication of neural implants. Using our nerve-on-a-chip platform, we evaluated the efficiency of this thin-film polymer to block neural conduction in peripheral nerves.

## Results

### Nerve-on-a-chip design and recording capability

**Fig. 1:**
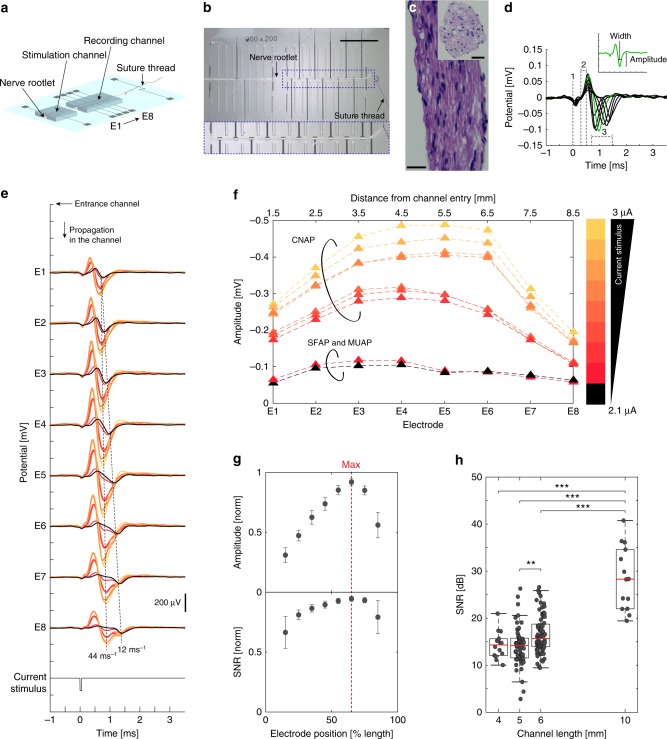
Nerve-on-a-chip design and recording capabilities. **a** Schematic of the nerve-on-a-chip platform. **b** Photographs of a teased nerve rootlet (100 μm diameter) inserted in the platform. Recording and stimulation electrodes are 100 × 300 μm^2^ and 100 × 600 μm^2^, respectively; electrode pitch is 1 mm; stimulation and recording microchannels are 8 and 10 mm long respectively. One end of the rootlet is tied with a suture thread then pulled inside the channel, as depicted in **a**. Scale bar: 5 mm. **c** Hematoxylin staining of cross and longitudinal section of a rootlet. Scale bar: 25 µm. **d** Superimposed recording of one SFAP along eight electrodes, highlighting stimulation artifact (label #1, simultaneous on all electrodes), onset artifact provoked by SFAP entry in the recording microchannel (label #2, simultaneous on all electrodes), and biological signals (label #3, depolarization wave, delayed between each electrode) (stimulation current: 2.1 µA, phase: 50 µs). Insert: SFAP amplitude is measured from baseline and SFAP width is measured at half amplitude. **e** Representative nerve signals recorded along one rootlet by all eight electrodes with increasing stimulation current (increment: 0.1 µA). Recorded signals are either SFAP, MUAP, or CAP. Color from black to yellow: stimulation current from 2.1 to 3 μA (increment: 0.1 μA, phase: 50 µs). **f** Maximal amplitude of neural signals along the channel. Increasing current pulses trigger SFAP, MUAP, then CAP (color code as in **e** and **f**). **g** Average of normalized SFAP amplitude and SNR along all eight electrodes. Maximal SNR and amplitude is reached at 2/3 of the microchannel length (red dashed line). Error bar: pooled standard error (*n* = 14 SFAP, each repeated 10 × ). **h** Boxplots of SFAP SNR recorded in microchannel. SNR is positively correlated with velocity (Supplementary Fig. 3); resulting SNR range increases with microchannel length. Pair comparison was done using Kruskal–Wallis test (α = 0.05) with Bonferroni correction (4 mm: *n* = 22, 5 mm: *n* = 75, 6 mm, *n* = 86 10 mm: *n* = 16). Significance: ***p < 0.001, **p < 0.01

The nerve-on-a-chip platform consists of two aligned microchannel electrodes prepared on a glass carrier through which a nerve rootlet is threaded (Fig. 1a). The polydimethylsiloxane (PDMS) microchannels have a section of 100 × 100 μm^2^ and a 4–10 mm length. Platinum microelectrodes are distributed along the microchannels—two electrodes in the stimulation channel, and up to eight electrodes in the recording one (with 100 × 300 µm^2^ active sites). The complete fabrication of the nerve-on-a-chip platform (to final packaging in a Petri dish) can be carried out within 2 days and with multiple electrode layouts. Rat nerve rootlets are dissected from the explanted spinal cord, which provides excitable roots for about 6 h post-mortem. Nerve roots are dissected into strands of about 100 μm diameter and 2–4 cm length (Figs. 1b, c, Supplementary Fig. 1). Next, a nerve rootlet is delicately pulled through both stimulation and recording microchannels and kept at 37 °C. Explanted nerve roots are kept on ice in Hibernate A until further dissected so that recordings from tens of teased rootlets can be completed within a single day of experiment. For example, from an L5 root dissection, 10–15 rootlets are obtained and show a 90% excitability within the first 2–3 h. Beyond this time window, slow component of MUAP or CAP become more difficult to record, suggesting thinner fibers degrade faster than larger ones. On average, each rootlet is used for about 10 min in the nerve-on-a-chip thus does not require perfusion.

Upon electrical stimulation in the first microchannel (µA range, 5 µs cathodic pulses), action potentials were triggered within the nerve rootlet. Figure 1d illustrates a traveling SFAP recorded by each of the eight electrodes distributed along the recording microchannel. Confinement of the nerve rootlet in the insulating microchannel substantially amplifies extracellular signals^28^ (Supplementary Note 1). Following the stimulation artifact (#1 label), the SFAP propagates along the axon in the recording microchannel (#3 label). The initial onset (#2 label) is an artifact caused by the SFAP entering the microchannel^28^. Using one nerve rootlet and gradually increasing the stimulation current from 2.1 to 3 μA, SFAP, MUAP, then CAP were elicited thereby providing a variety of nerve signals, similar to those occurring in vivo (Fig. 1e). Large nerve fibers displayed the lowest stimulation threshold; increasing the stimulation current dominantly recruited the largest fibers contained in the rootlet^13^. Signal conduction velocity was next computed from spike propagation delay recorded between two neighboring electrodes (1 mm apart). At low stimulation current (2.1 and 2.3 µA), fibers with distinct conduction velocities (and therefore distinct axon diameter)^40^ were recruited (Fig. 1e: 44 and 12 ms^–1^). At higher stimulation currents, fibers with the highest conduction velocity (44 ms^–1^) were mostly recruited, thereby forming a CAP. The CAP amplitude further increased with the stimulation current (Fig. 1f).

In order to quantify the amplification along the channel, SFAP were obtained using the minimal threshold of stimulation current eliciting a single-neural response. When, the minimal current elicited two spikes, the data were discarded. We observed that the amplification of both the SFAP amplitude and SNR varied along the channel length and were the highest at the location two-thirds of the channel length (Fig. 1g). Increasing the microchannel length further increases the signal amplitude and its SNR (Fig. 1h) and asymmetry of the peak amplitude down the microchannel (Supplementary Fig. 2). This is consistent with theoretical analysis published by FitzGerald et al.^28^ and gives an indication of the direction of propagation of the neural signals.

### VSR

**Fig. 2:**
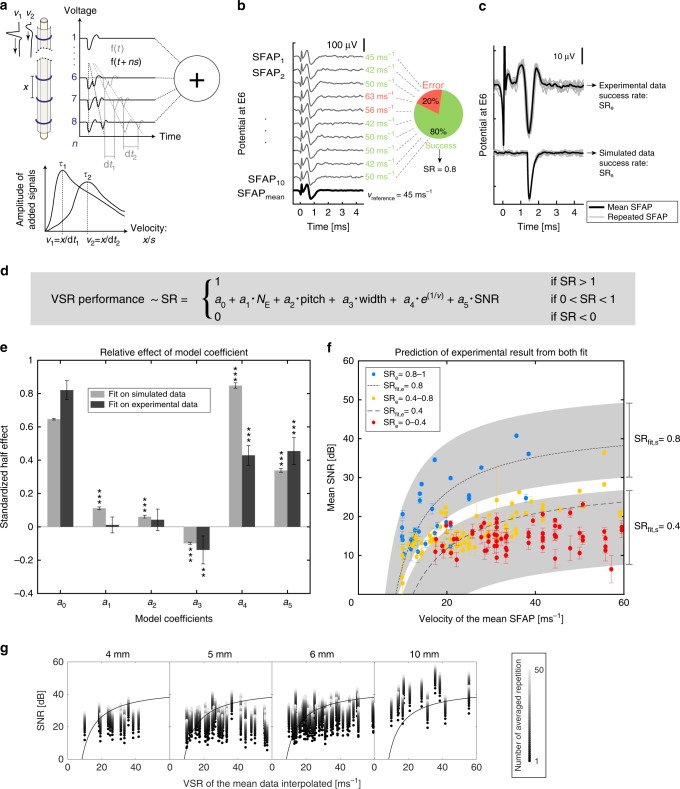
Prediction of VSR algorithm performance. **a** VSR principle, adapted from^24^. SFAP are traveling along the nerve recorded by *n* electrodes spaced by *x* (gray line). The recordings on each electrode are shifted by *ns* (black line), where *s* corresponds to the propagation delay between two electrodes (multiple of the sampling frequency). For each value of *s*, all recordings are summed. The sum becomes constructive when *s* matches d*t* (alignment of propagating SFAP). **b** Example of calculation of the SR. Each simulated or recorded SFAP is elicited 10 times and averaged. The SR is the proportion of SFAP with a calculated velocity equal to the velocity of the mean SFAP (see Eq. 1). **c** Example of recorded SFAP repetitions and mean (experimental data) and its simulated version (amplitude = 55 µV, SNR = 30 dB, width = 0.22 ms, and velocity = 13.3 ms^–1^). **d** Linear model resulting from preliminary simulation (see Supplementary Methods). The model terms are function of the number of electrode (*N*_E_), the electrode pitch (pitch), the SFAP width at half prominence (width), the SFAP velocity (*e*^1/*v*^) and the maximum SNR along the channel (SNR). The model coefficients *a*_i_ were fitted to experimental and simulated data. **e** Relative effect of each model terms on the SR. Model coefficients, expressed as standardized half effect, were computed from simulated and experimental data. Error bar: 95% confidence interval. Significance (ANOVA, α = 0.05, Supplementary Methods 3): ***p < 0.001, **p < 0.01. **f** Experimental SR (SR_e_), as well as SR predictions from our model fitted on experimental (SR_fit,e_, dashed line, for width = 0.03 ms, *N*_E_ and pitch: n/a) and simulated data (SR_fit,s_, gray area, width = 0.03, *N*_E_ = 3-8, pitch = 1.33–0.67 mm). Error bar: standard deviation (*n* = 10). **g** SR as a result of SNR, velocity and channel length. Black dot shows experimental results and lighter dots show the calculated increase of SNR in function of signal averaging. The black line (SR_e_ = 0.8) delimits the region for “safe” velocity calculation (above line)

The nerve-on-a-chip platform supports more than one electrode per microchannel thereby opening an opportunity to register-rich axonal information such as signal propagation direction and velocity. We implemented the VSR algorithm described by Donaldson et al.^41–44^ to compute the direction and velocity of nerve signals within the microchannel (Fig. 2a). Neural signals recorded by each electrode along the microchannel are shifted by an artificial time delay then summed. When the artificial time shift matches a neural signal propagation delay, the signals add up constructively, and the detection of the maxima can be used to compute the signal velocity. We used a systematic approach to characterize neuron signal propagation along microchannels of different dimensions (10 different designs) and we implemented a computational model that simulates these signals. Next, we built and validated a model that predicts the error rate of velocity calculation as a function of the geometry of the microchannel and signal waveform.

SFAP were recorded with the nerve-on-a-chip platform then processed with the algorithm described above. We used the success rate (SR) to quantify the algorithm performance, defined as the proportion of successfully calculated velocity within 10 repetitions of an SFAP (Fig. 2b, thin lines). The reference velocity (*v*_reference_) was defined as the velocity computed from averaged repetitions (Fig. 2b, thick line). Ten platforms with varying microchannel length, electrode number, and electrode pitch were fabricated and enough SFAPs were recorded so that the full range of signal amplitude, width, and velocity was obtained for each design (Supplementary Fig. 3).

First, we used experimental data to measure the amplification factor along microchannels with different length (4, 5, 6, and 10 mm, Supplementary Fig. 2) and further implement a computational simulation of SFAP propagation (Fig. 2c) to deduce SR model terms and functions shown in Fig. 2d (see Supplementary Methods for complete development). We found that (i) the SNR rather than the signal amplitude affects the SR, (ii) the velocity is exponentially correlated to the SR, and (iii) the microchannel length, number of electrodes, electrode pitch, and SFAP SNR and width are linearly correlated to the SR (Supplementary Fig. 4).

Next, we simulated propagating SFAP along the microchannel (see Supplementary Methods for complete development) and compared them with experimental fits. We computed the influence of each parameter expressed as relative half effect on the SR (Fig. 2e). Although all terms significantly affect the SR in simulations, the number of electrodes and pitch have no significant effect in experimental conditions. As the eventuality of an over fit was discarded (Supplementary Fig. 5), we hypothesize this difference depends on experimental variability. Indeed, although in simulation data, there is no change in waveform across repetitions of an SFAP, irregularities are observed during and across recording sessions. In both experimental and simulated analyses, we found the SR to be mostly sensitive to the signal velocity and the SNR (Fig. 2f). Both sets of coefficients predict experimental measures of the SR (SR_e_) with a similar precision (Fig. 2f, Supplementary Fig. 6). The length of the recording microchannel determines the SNR range at which SFAP are recorded (Fig. 1f) and defines the maximal value of the SR for velocity computation. The longer the microchannel (e.g., > 10 mm), the higher the SR ( > 0.8). Shorter microchannel requires SFAP averaging to increase both the SNR and the SR (Fig. 2g, fitting details in Supplementary Methods).

### Heat-induced neuroinhibition upon P3HT:PCBM illumination

**Fig. 3:**
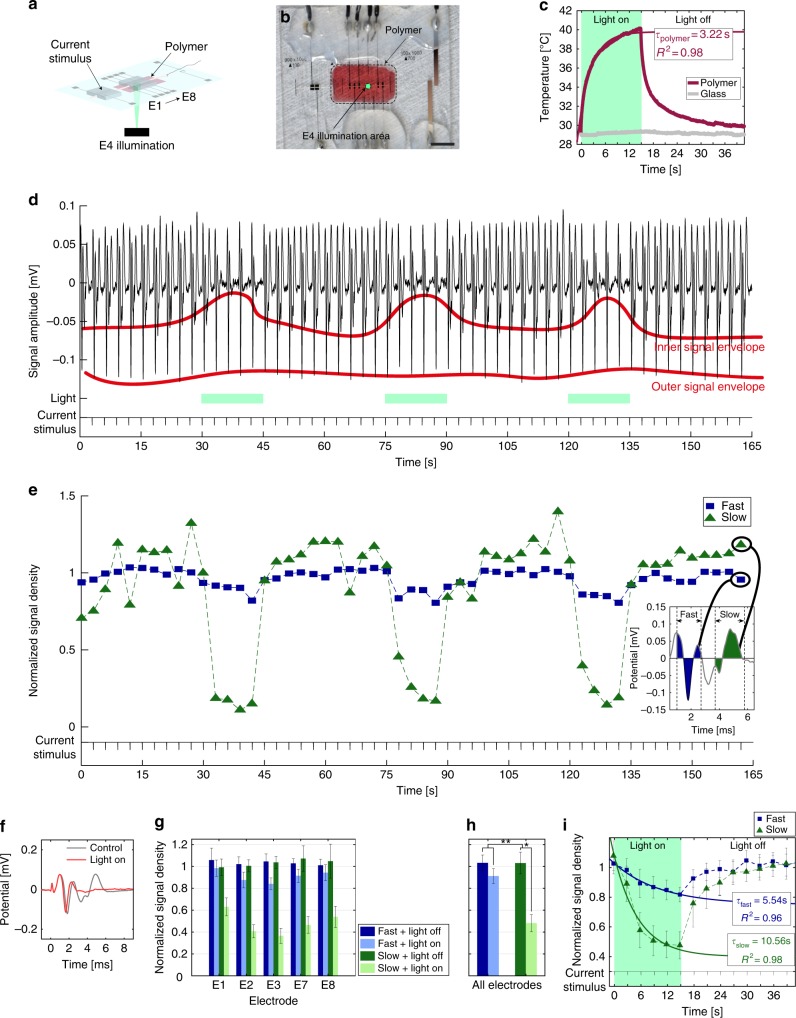
Heat-induced neuroinhibition. **a**, **b** Schematic and photograph of the nerve-on-a-chip integrating a thin film of P3HT:PCBM on the floor of the channel (red). Illumination is focused on electrode E4. Scale bar: 5 mm. **c** Temperature during illumination. A 15-s light pulse was applied through the polymer and bare glass. The temperature changed with a time constant of 3.22 s (Supplementary Equation 13). **d** Close-up of a rootlet concatenated recording captured downstream the illumination area (electrode E7). Every 3-s MUAP were elicited with a current pulse. Variation in signal envelope shows reduction of the signal amplitude. **e** NSD of slow and fast fibers corresponding to the recording in **d**. Box: a representative MUAP; the fast and slow signals were integrated to calculate their corresponding NSD. Each NSD was then normalized with its control response (10 first elicited MUAP). **f** MUAP captured by electrode E7 and averaged across light pulses. **g** NSD average across all four rootlets, repeated stimulation (3 × ) and light treatment at each Pt electrodes (3 × heating/cooling cycle). Right-panel: additional average across Pt electrodes are shown in the right-panel. Error bars: pooled standard error (*n* = 4, each repeated 9 × ). **h** Additional average from data in **g** across Pt electrodes. Error bars: pooled standard error (*n* = 4, each repeated 45 × ). A four-way ANOVA was performed (α = 0.05, Supplementary Methods). The illumination effect is significant, as well as the difference of inhibition between fast and slow fibers, meaning that the effect of the illumination was stronger on slow fibers than on fast fibers. Post-hoc one-way ANOVA (α = 0.05, Supplementary Methods) applied on slow and fast fiber separately showed a significant effect of the illumination on slow but not on fast fiber. Significance: **p < 0.01, *p < 0.05. **i** Kinetics of inhibition of slow and fast fiber averaged across light pulses, Pt electrodes and rootlets. Exponential fit (Supplementary Equations 15-16) highlights a higher time constant for slow fiber than for fast fiber. Error bars: pooled standard error (*n* = 4, each repeated 60 × ). *R*^2^ R-squared of the fit

We adapted the microfabricated nerve-on-a-chip platform to integrate optically transparent indium tin oxide (ITO) electrodes within the recording microchannel thereby allowing for concurrent optical stimulation and neural signal recording. A thin film of P3HT:PCBM polymer was coated at the floor of the recording microchannel (Figs. 3a, b). Shining green light (1 mm diameter spot size, irradiance 16 mW mm^–2^, 510 and 550 nm wavelength) through the thin blend film induces local surface heating (Fig. 3c, Supplementary Fig. 7, Supplementary Movie 1) with time constant of *τ*_polymer_ of 3.22 s.

Representative neural signals (recorded at electrode E7) from a nerve rootlet threaded through the modified nerve-on-a-chip are shown Fig. 3d. Current stimulation (10–35 μA, 50 μs pulse width, 0.33 Hz) elicits MUAPs. Two superimposed signal envelopes are clearly visible in the raw data (Fig. 3d, orange lines). Illumination cycles (15-s light pulse) are applied to the nerve rootlet within the recording microchannel and through the electrode E4. We observe a visible reduction of signal amplitude during the illumination phase (Fig. 3d). This appears in signals recorded by all electrodes within the microchannel and is more pronounced in the inner signal envelope than in the outer one. As neural signal amplitude in the microchannel is positively correlated with signal velocity^26^, these results suggest a stronger inhibition of MUAP amplitude in the slow fibers. As spike amplitude can sum up or cancel out depending on their relative position in the microchannel and are not distinguishable on all electrodes, we used the normalized signal density (NSD) to quantify signal inhibition rather than MUAP amplitude. We integrated and normalized the fast and slow fiber activity (Fig. 3e, f). Integration intervals for each electrode are shown in Supplementary Fig. 9. Calculation was done based on a velocity threshold of 5 ms^–1^ (calculation details in Supplementary Methods). Then, we distinguish slow (green data, conduction velocity < 5 ms^–1^) from fast (blue data, conduction velocity > 5 ms^–1^) fibers. NSD averages from recordings collected from four distinct rootlets (Supplementary Fig. 8a) are displayed in Fig. 3g. We discard recordings from ITO electrodes (E4–E6) as a significant increase in white noise was observed close to the illumination spot (Supplementary Fig. 8b). The statistical analysis is detailed in Supplementary Methods. We observe maximal inhibition of both fast and slow fiber close to the illuminated area (electrode E3). The inhibition gradually decreases as the distance from the illuminated area increases, suggesting that photo-induced heat is diffusing. To differentiate MUAP inhibition from MUAP conduction blockade, we consider recordings from electrodes positioned downstream the illumination area, i.e., electrodes E7 and E8. NSD of fast fiber fully recovers to 1, whereas NSD of slow fiber remains inhibited, suggesting fast fibers are locally inhibited by local heating while slow fibers appear permanently blocked (Fig. 3g). The heat has a significantly stronger effect on slow fibers than on fast fibers (Fig. 3h).

Illumination of the dry semiconductive blend leads a temperature increase localized to the spot size (Supplementary Movie 1, Supplementary Fig. 7). In the nerve-of-chip platform, the induced heat also diffuses through the extracellular medium and the rootlet thereby inducing some inhibition up- and downstream the illumination spot. An increase in temperature affects signal propagation at different levels. First, voltage-gated sodium channel activation and inactivation kinetics is accelerated, which in turn shortens the action potential, decreases its amplitude, and increases conduction velocity^45–47^. This is visible in Supplementary Movie 2 (observing propagating action potentials from fast fibers). Second, heating hyperpolarizes the cell membrane thus competes against the depolarization wave forming the action potential. Heat-induced hyperpolarization decreases membrane resistance and increases membrane capacitance^48^. As both membrane resistance and capacitance are functions of nerve fiber diameter, it is expected the hyperpolarizing effect will differ according to the fiber diameter. NSD kinetic of slow and fast fibers with light-induced heating is illustrated by “discharging/charging” like profiles (Fig. 3i). The corresponding time constant *τ* of the fast fibers is nearly half that of the slow fibers, and the NSD value at the end of the heat/illumination cycle of the fast fibers is nearly twice that of the slow fibers. This confirms slow fibers inhibition to light-induced heating is stronger than that of fast fibers. The activity from both fibers groups recovers their initial level once illumination is turned off. Averaged NSD for the three heating/cooling cycles led to no significant difference in the recovery phase, indicating that the thermal inhibition is reversible (Supplementary Fig. 11).

## Discussion

Our nerve-on-a-chip platform provides significant amplification of extracellular axonal signals. Microchannel restricts the extracellular space at the electrode—tissue interface increases its impedance, which results in an increase in extracellular signals^28^. Explanted nerve rootlets were used to demonstrate that signal sensitivity can be increased by either decreasing the cross-sectional area or by increasing the length of the microchannel in previous studies. Microchannels were also used for in vivo recording of regenerating fibers with a cross-section allowing nerve growth and vascularization: 100 × 100 µm^2^^33,34,36,49^. Similarly, using microchannels as a stimulating cuff reduces the necessary current to elicit nerve fiber response to the µA range^26^. Optimal electrode size is a trade-off between recording selectivity and thermal noise^50^. In previous studies, 100 × 300 µm^2^ electrodes enabled recording of SFAP in vivo^34^.

In this ex vivo study, we maximized the length of both stimulation and recording microchannels according to tissue length. We separated recording and stimulation channels to further decrease stimulation artifacts. We used microchannel and electrode dimensions compatible with in vivo regeneration to show the recording potential of the optimized microchannel. In 10 mm long microchannel, we observed the all-or-none principle as increasing the current lead to an incremental increase of the amplitude. The relationship between amplitude and velocity was consistent with previous studies on SFAP recording^26^. However, over 16 recorded SFAP, 3 outliers (with higher amplitude than expected for a given velocity) were obtained as well. Thus, we cannot exclude that, occasionally, a detected waveform is the result of the superposition of two identical signals traveling at the very same velocity (Supplementary Fig. 3b). For experiments relying on SFAP recording, one preliminary explant should systematically be devoted to calibration recording. The distribution of amplitude-velocity (as a function of microchannel length and section) would further be used as a reference to discard non-SFAP data.

In order to implement a VSR in a regenerative implant for in vivo applications, the length of a microchannel implant should be as long as possible to maximize the SNR but short enough to sustain nerve regeneration. Six-millimeter length appears as a good trade-off. The number and spacing of electrodes have a negligible impact on the reliability of velocity calculation in millimeter length microchannels, however, the minimal number of electrodes to run the VSR algorithm is 3. As the smallest spikes are not systematically visible at both ends of the microchannel, four electrodes should enable to systematically record spikes at three sites. In the regenerative implants, spike sorting and averaging (at least 50 repetitions) should be done prior to applying the VSR for velocity up to 35 ms^–1^. For superior velocity, reliability of SFAP velocity should be assessed individually by calculating the SR. Finally, all SFAP with a low SR should be discarded from in vivo analysis.

Different strategies to optimize the VSR algorithm performance, based on qualitative analysis, have been previously implemented in proof-of-principle studies^44,51,52^. Our platform enabled rapid data collection and easy variation of the recording design in a cost-efficient manner. Thanks to our data library, our simulation and experimental validation enabled calculation of velocity measurement range as a function of the calculation error rate, the SFAP waveform and the desired implant geometry. Although some of the waveforms might have been superimposed spikes, it did not influence our SR model, as the amplitude and velocity are independent variables. Classification algorithm often requires the experimenter to estimate the thresholds to detect spikes (root mean square – based threshold) or differentiate them (principal component analysis) to minimize false-positive and -negative signal detection. Our platform enables to elicit single-cell spike in a timely controlled manner, thereby providing direct read-out. Consequently, we were able to characterize not only the error rate of velocity calculation but also quantify on which variable it depends. To our knowledge, it is the first characterization of classification performance done in a dish. Our nerve-on-a-chip model can offer more insight on optimizing decoding algorithm for neuroprosthetic application.

Although heat inhibition was observed, it is unlikely that photo-thermal thin films can be used to efficiently silence whole-nerve activity as conduction block was not observed in the largest myelinated fibers, even with a temperature increase of 10 °C. However, compromising for inhibiting antidromic stimulation, selective inhibition toward thinner fibers provides an exciting opportunity for pain relief. As pain fibers have the smallest diameter, and heat diffused over a few millimeters within the microchannel, the semiconductive blend film could be integrated in a nerve implant to selectively block pain without affecting other nerve functions. In situ light stimulation could be achieved with flexible optical fibers^53,54^ or micro-light-emitting diodes embedded in the implant wall^55^. This approach would bypass the need for genetic modification to control pain fibers or the use of pain inhibiters^56^. Moreover, the patterning of the photo-thermal semiconductive blend within the nerve implant could enable a treatment localized in space and time to affected neurons to treat chronic or phantom pain.

We used our platform to spatially control the propagation of neural signals and were able to refine conclusions from a previous study^4^ by adding the effect of fiber size to the outcome of silencing. We confirmed that heat indeed inhibits neural activity but not on large myelinated fibers. Investigation of pain suppression often relies on in vivo indirect read-out such as Von Frey testing as it is challenging to predict selective inhibition. Our results show the importance of controlling tissue heterogeneity in vitro and how these tools can extend to applications in the field of neuroprosthetic research.

The microfabricated nerve-on-a-chip platform is a versatile in vitro tool to study and quantify peripheral nerve electrophysiology including nerve signal density and kinetics of activity change of myelinated fibers. The platform offers a good compromise between SNR, stimulation selectivity, and temporal selectivity of recordings. In addition, the ex vivo model reflects accurately the heterogeneity present in peripheral nerve tissue and enables advanced analysis based on the full range of SFAP waveforms rather than on a single nerve fiber type. Of note, the nerve-on-a-chip platform described here answers efficiently the 3 R principle. We built our neural signals library in 10 days of experiment and acquired the neuromodulation data in 2 days, involving 12 animals in total and a minimal degree of severity procedure.

The nerve-on-a-chip platform is an efficient tool for designing and evaluating new implantable electrodes: a range of electrode configurations and materials can be tested to optimize implants geometry, define electrical and optical powering of neural interfaces, validate neuromodulation strategies and selectivity, or advance spike sorting algorithms. We foresee the ex vivo platform may also find applications in neurotoxocity testing where alternatives to expensive and time consuming in vivo testing are needed.

In summary, the microfabricated nerve-on-a-chip design can be tailored to rapidly achieve complex multifactorial analysis, computational modeling and experimental validation while delivering a robust statistical analysis. Future improvements of the nerve-on-a-chip platform include reducing of the cross-section area of the microchannel to further amplify the extracellular signals, perfecting nerve root teasing, and extending tissue survival with improved conservation environment. In addition, data library based on MUAP and corresponding algorithms could be developed to also extract their composing SFAP.

## Methods

### Device fabrication

The standard nerve-on-a-chip platform is made of two stimulation electrodes, eight recording electrodes (pitch: 1 mm), and a reference and grounding electrode. All tracks and electrodes were made in platinum. Glass wafers were cleaned in a piranha solution. A Ti/Pt thin film (25/200 nm) was evaporated and patterned on glass during a lift-off process (positive photoresist: LOR/AZ1215 800/1200 nm). Glass wafers were diced into rectangular chip of 4 × 2.5 cm^2^. PDMS microchannels with a 100 × 100 μm^2^ cross-section were obtained through soft photolithography; silicon master molds were obtained by BOSH RIE, silanized and double casted with PDMS. Recording electrodes were encapsulated in a PDMS microchannel (10 mm length) thereby defining 100 × 300 μm^2^ electrode contacts. Similarly, stimulation electrodes were encapsulated in a 8 mm PDMS microchannel leading to 100 × 600 μm^2^ electrode contacts. Connector wires were soldered to the chip and encapsulated in silicone glue. The chip was glued inside a polystyrene well.

For the VSR experiment, 10 platforms with different recording configuration (channel length, number of electrode, and electrode pitch) were produced. For the polymer experiment, stimulation electrodes were enlarged to 100 × 900 μm^2^ and central recording electrodes were made of ITO to allow light transmission (E4, E5, and E6): ITO electrodes (200 nm) were patterned (AZ1512 1200 nm) above platinum tracks and etched using HCl. In addition, the semiconductive blend was spin coated on top of the recording electrode. P3HT and PCBM were individually dissolved in chlorobenzene anhydrous (concentration of 20 mg mL^–1^) and the obtained solutions stirred overnight at 70 °C. Subsequently, the solutions were filtered (PTFE filters, 0.45 µm) and mixed in a 1:1 volume ratio. Plastic tape was used to restrict the area to be coated. The chip surface was activated by oxygen plasma. The polymer (50 µL) was spin coated on the electrode layer at 1000 rpm for 60 s and baked 2 h at 80 °C (nominal thickness of 200 nm).

### Tissue extraction

All animal procedures and experiments were approved by the Veterinarian Offices of the Cantons of Vaud, Switzerland. Ten and three adult male Lewis rats were used for the VSR and photo-thermal case studies, respectively. They were provided ibuprofen in drinking water for 12 h and anesthetized with isofluorane. The spinal cord was exposed via a long laminectomy (T12–L6) and longitudinal dura matter incision. Nerve roots were cut at the exit foramen and the spinal cord with attached roots was left on ice in Hibernate A medium (Life Technology) until further dissected. The dissection and recording took place in Hanks’s balanced salt solution kept at room temperature. Nerve roots were cut from the spinal cord to obtain a nerve strand of several centimeters. They were teased into rootlets with a diameter of 100 μm (Supplementary Fig. 1). One end of the rootlet was tied with a nylon suture (Ethicon 9.0), used to pull it inside the microchannels. For testing recording capabilities and for the VSR experiment, nerve activity was recorded from ventral L5–L4 root at 37 °C using a hotplate. For the polymer experiment, nerve activity was recorded from dorsal S1 root at room temperature.

### Histology

Dissected rootlets were fixed in 4% paraformaldehyde overnight at 4 °C. Tissues were embedded in paraffin and sliced with 1.5 µm thickness. Hematoxylin was used for tissue staining; imaging was performed with bright field microscopy.

### Data acquisition

Each recording electrode was connected to an AC amplifier (AM-system 1700) with a gain of 1000 and a band pass filter of 100Hz–5kHz (–40 dB decade^–1^). Data were acquired using Micro-1401 hardware and Signal software (CED). Sampling frequencies varied with the experiments: for testing recording capabilities and for the VSR experiment, data were recorded at a sampling frequency of 50 kHz and further interpolated (cubic spline, MATLAB) at 500 kHz. For the polymer experiment, data were recorded with a sampling frequency of 25 kHz and no further interpolation was made. Finally, all recordings were band filtered using (butterworth, order = 1, MATLAB) with cutoff frequency of 100 Hz and 3 kHz.

### VSR

Our nerve-on-a-chip platform is depicted Figs. 1a, b. We fabricated different designs in order to vary experimentally the SNR range (via the recording microchannel length), the number of electrode and the electrode pitch (summarized in Supplementary Table 1). Ten adult male rats were used in this study. With each platform design, we elicited and recorded 30 different SFAP and each SFAP was elicited 10 times using minimal threshold current stimulation (square, cathodic, phase: 50 µs). We built a custom software in MATLAB to supervise spike detection in the individual spikes and in the average of spike repetition. The software then extracted all the biological parameters, VSR results and performance on all data. The ladder was defined as follow:

1 $${\mathrm{Performance}}\sim {\mathrm{SR = }}\frac{{N_{{\mathrm{SFAP}}} \in [v_{{\mathrm{reference}}} + \delta ;v_{{\mathrm{reference}}} - \delta ]}}{{N_{{\mathrm{repetition}}}}}$$

where *N*_SFAP_ is the count of SFAP velocity successfully calculated, *N*_repetition_ is the number of repetition of an SFAP, *v*_reference_ is the calculated velocity of the mean SFAP, *δ* was set according to the sampling step of the velocity at 50 ± 5 ms^–1^. SFAP with a velocity above 60 ms^–1^ or containing multiple spikes were discarded from the study.

In order to model recorded data, we extracted several parameters from the experimental setup that we included in our simulation and reproduced the same data processing step. (Details on the implementation of the model, simulation parameter, and model fitting can be found in Supplementary Methods). First, we measured SFAP amplification for each channel length; we normalized the SFAP amplitude along the channel and averaged the recordings. The same analysis was done on the SNR and the SFAP width (Supplementary Fig. 2). Second, we measured biological range of amplitude, width, SNR at mid-channel (Supplementary Fig. 3a) and how they are related to the velocity (Supplementary Fig. 3b). We simulated SFAP with all possible combination of width, SNR, and velocity and included channel amplification factors corresponding to each nerve-on-a-chip design. Each SFAP was simulated 10 times to enable the measurement of the SR. We used intermediate simulation to implement a linear model correlating the SR with SFAP width, velocity, SNR, the number of electrode, and electrode pitch (see Supplementary Methods). It was fitted to both simulated data and experimental data to calculate the model coefficients.

### Heat-induced neuroinhibition upon P3HT:PCBM illumination

Four nerve rootlets (dorsal S1) were placed in the chip coated with the polymer and mounted under a light source (1 mm diameter spot size, irradiance 16 mW mm^–2^, wavelengths 510 and 550 nm) at room temperature. Each rootlet was stimulated using current (square, anodic, phase: 50 μs, pulse frequency: 0.33 Hz). Every 45 s, a 15-s light pulse was illuminating the electrode E4. Signals envelope and NSD for slow and fast fiber were computed for each root at each electrode. A factorial analysis of variance (ANOVA) was used to analyze the effect of the light on fiber type at each electrode (see Supplementary Methods for detailed statistics). We quantified the heating upon illumination with an IR camera. We applied an illumination pulse with the same duration and intensity as in the recording experiment on plain glass and glass coated with the blend.

## Electronic supplementary material

Supplementary Information

Description of Additional Supplementary Files

Supplementary Movie 1

Supplementary Movie 2

Supplementary Movie 3

## Data Availability

The data that support the findings of this study are available from the authors on reasonable request.
