# Supplementary material for: A microfabricated nerve-on-a-chip platform for rapid assessment of neural conduction in explanted peripheral nerve fibers
Source: Nat Commun. 2018 Oct 23;9:4403. doi: 10.1038/s41467-018-06895-7 (PMC6199302; doi:10.1038/s41467-018-06895-7)
Supplement: Supplementary file 1 — Supplementary Information [file 41467_2018_6895_MOESM1_ESM.pdf]

Supplementary Information

A microfabricated nerve-on-a-chip platform for rapid assessment of neural conduction in explanted peripheral nerve fibers

Gribi et al.

Supplementary Figures and Tables

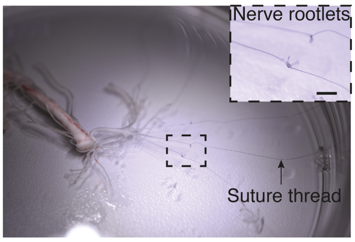

Supplementary Figure 1 — Dissection of adult rat spinal cord. Photograph of an explanted spinal cord from T13 to S1 segment and its attached spinal roots. Scale bar: 1 mm.

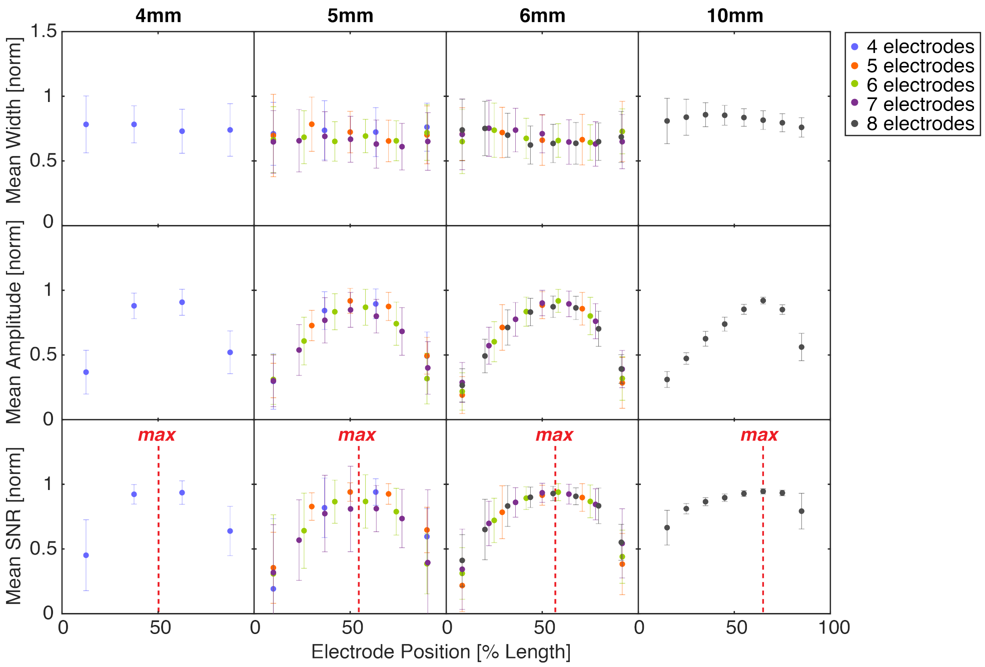

Supplementary Figure 2 — Amplitude, SNR and width along the channel. SFAP with velocities between 0 and 60 ms^{-1} were recorded and each repeated 10 times. To measure the amplification, we normalized the SFAP amplitude along the channel and averaged the recordings. The same analysis was done on the SNR and the SFAP width. Recordings were done in channel with different length (4-10 mm). SFAP were propagating from 0 to 100 % of the channels length. For 5 and 6 mm, the number of electrode and spacing varied as indicated. Estimated position of maximum SNR for each channel length is indicated with a red dashed line. Error bars: pooled standard error. 4 mm: n = 22, 5 mm / 4 electrodes: n = 16, 5 mm / 5 electrodes: n = 23, 5 mm / 6 electrodes: n = 19, 5 mm / 7 electrodes: n = 17, 6 mm / 5 electrodes: n = 15, 6 mm / 6 electrodes: n = 25, 6 mm / 7 electrodes: n = 24, 6 mm / 8 electrodes: n = 22, 10 mm / 8 electrodes: n = 16, each repeated 10x

Length [mm]	N_E	Pitch [mm]	#SFAP	E_{max}
4	4	1	22	3
5	4	1.33	16	3
5	5	1	23	3
5	6	0.8	19	3
5	7	0.67	17	5
6	5	1.25	15	3
6	6	1	25	4
6	7	0.83	24	4
6	8	0.71	22	5
10	8	1	16	6

Supplementary Table 1 — Platform design and number of recorded sample. Summary of ten platforms used in the VSR experiment and number of recorded SFAP per design (#SFAP). First and last electrode were placed 0.5 mm away from the channel ends in 4, 5 and 6 mm long channel. First and last electrode were

placed 1.5 mm away from 10 mm channel ends. 30 SFAP were recorded in each platform and signal with a velocity > 60 ms^{-1} or containing multiple spike were discarded. Maximal amplification was reached at electrode E_{max}.

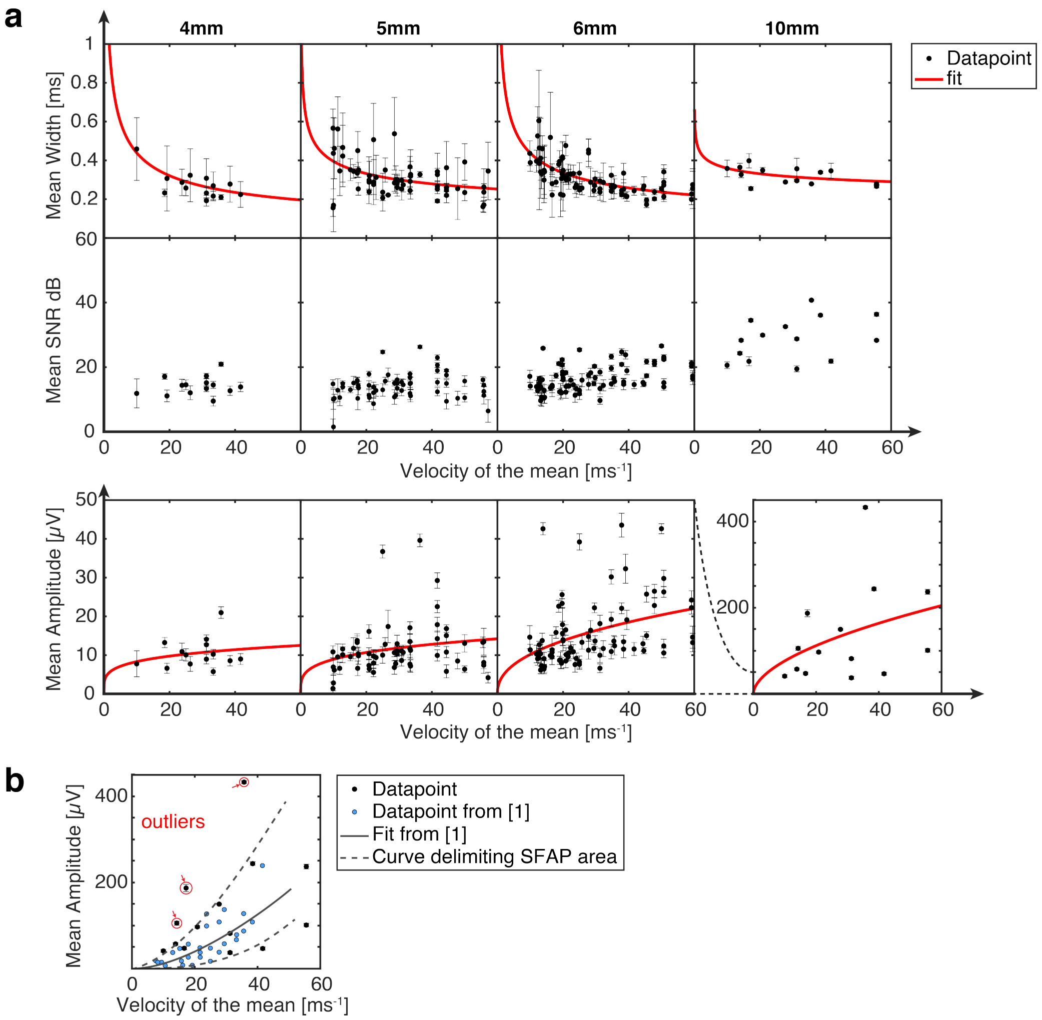

Supplementary Figure 3 — Amplitude, SNR and width across the channel. a, Ranges of width, amplitude and SNR mid-channel (extracted on the electrode giving the highest SNR, see Supplementary Table 1) were measured in correlation with the velocity. Width and amplitude are correlated to the velocity through a power function (Supplementary Table 2) [1]. We applied a fit accordingly. Error bar: standard deviation. **b**, Comparison of our data (black dots) with SFAP recording from *Fitzgerald et. al* (blue dots, adapted from[1]) for 10 mm long microchannels with a 10'000 µm^2 cross-section. The grey line shows the fit obtained in[1]. Dashed lines were added to delimit the area of amplitude-velocity inside which SFAP should lye. Most of our data is contained inside that area, suggesting that we obtained SFAP. Three outliers, probably containing superimposed spikes, are evidenced (circled in red). Note: the direct comparison of the amplitude was possible thanks to the filters being used. Figure error bars: standard error.

Fit	Length [mm]	*a*	*b*	R^2
width = av^b [ms]	4	1.24	-0.451	0.56
	5	7.03	-0.250	0.19
	6	1.05	-0.381	0.45
	10	4.74	-0.120	0.23
amplitude = av^b [µV]	4	4.94	0.227	0.05
	5	4.90	0.261	0.06
	6	3.78	0.431	0.14
	10	21.6	0.550	0.14

Supplementary Table 2 — Fit of SFAP width and amplitude as a function of velocity. Fits are shown in Supplementary Fig. 3a. Fitting constant: *a* and *b*, R-squared of the fit: R^2.

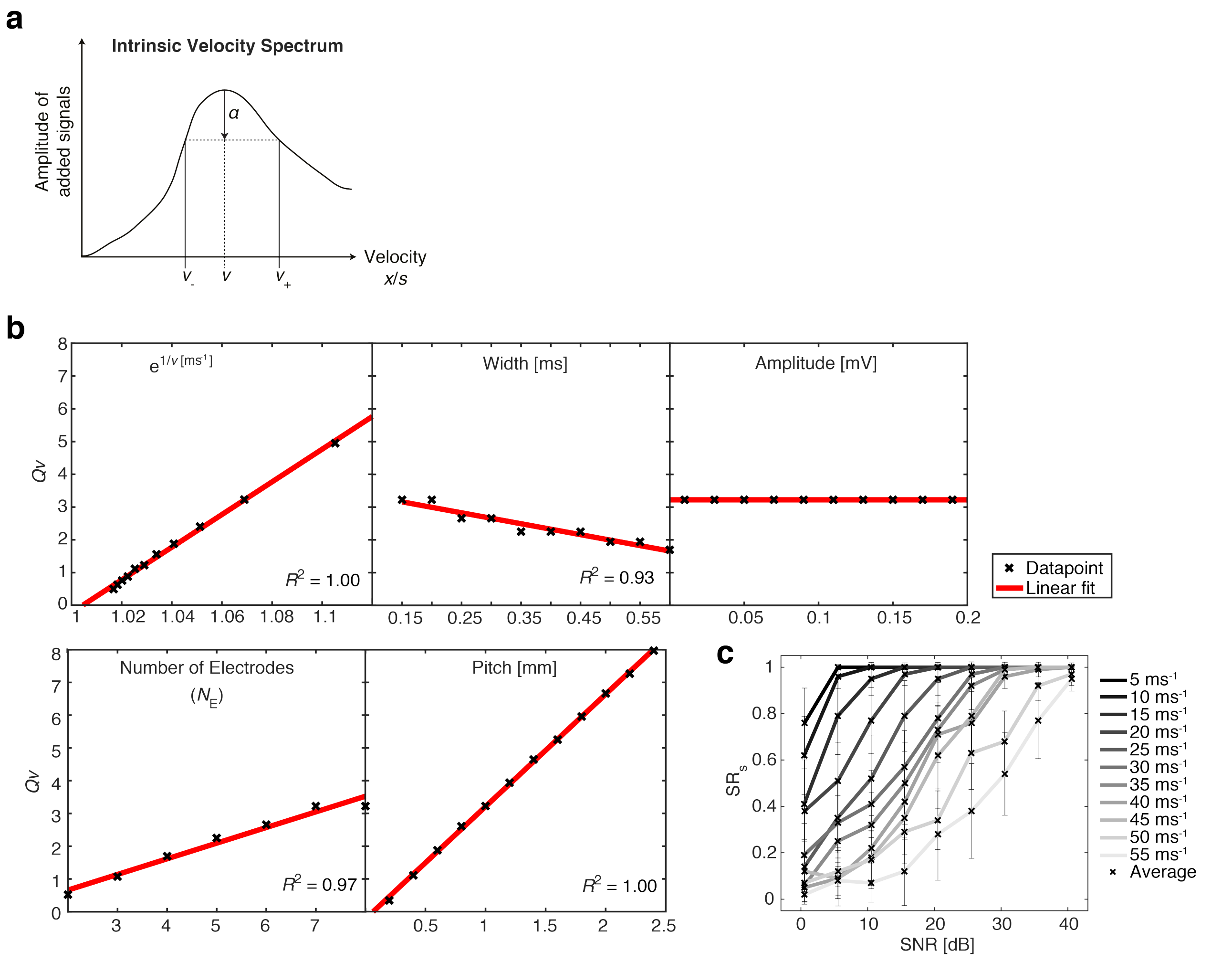

Supplementary Figure 4 — Model development. a, Illustration of the calculation of the velocity quality factor given in Supplementary Equation 2. v is the propagation velocity corresponding to the drawn IVS. v_+ and v_- are the velocity at which the IVS is attenuated of α. **b,** Results of Q_v calculation. Q_v was measured in function of AP amplitude, width and velocity as well as electrode number and pitch using simulated data (without added noise). The varying factor is indicated on the x-axis. When kept constant, values were set as followed: width = 0.15 ms, velocity = 15 ms^{-1}, pitch = 1 mm, number of electrode = 8. Q_v fit were done for each curve. The goodness of fit was calculated using the R-squared of the fit: R^2. Q_v is a linear function of $e^{\frac{1}{v}}$, width, amplitude, N_E and pitch (R^2 = 0.93 - 1.00). **c,** In order to measure the SR as a function of SNR, we simulated 10 repetitions of SFAP with added Gaussian white noise to mimic experimental data. We repeated the simulation in order to calculated the SR_s average of 10 trials. For each simulated SNR, we varied the velocity (5 - 60 ms^{-1}) in order to investigate potential interaction effect (between the SNR and the velocity). All other simulation parameters were kept constant with the same values as in a. Error bars: pooled standard error (n = 10, each repeated 10x).

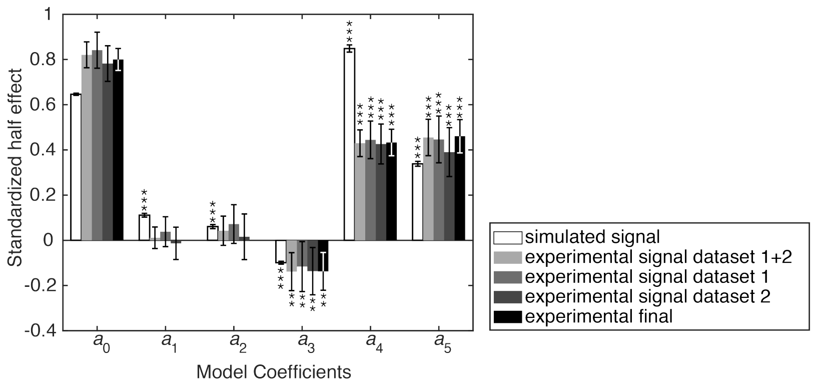

Supplementary Figure 5 — Standardized half effect of model coefficients. Coefficients, expressed as half effect, obtained with experimental data are different than with simulated data; the velocity is twice less influent and the effect of the pitch and the number of electrodes are not significant. In order to test if this difference was due to an over fit of the experimental data, we splitted the dataset in two and fit the model coefficients on each. As half-effects were similar in all case, we discarded the eventuality of an over fit. Error bars: 95 % confidence interval.

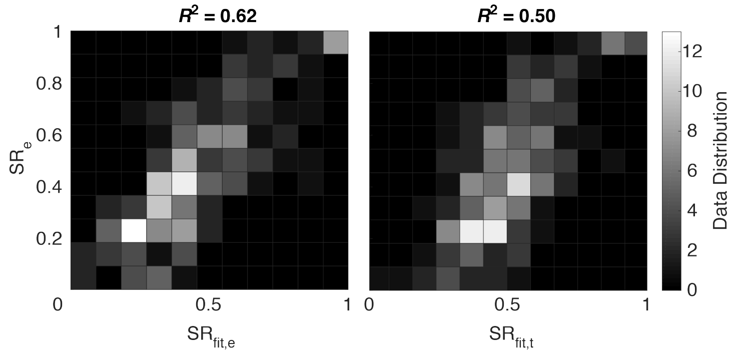

Supplementary Figure 6 — Goodness of fit between predicted and measured data. The experimental success rate (SR_e) was compared to prediction obtained with our SR model using the R-squared of the fit: R^2. Using coefficients a_i rising from model fit of experimental data lead to a better prediction ($R^2 = 0.62$) than using the coefficients obtained through a fit of simulated data ($R^2 = 0.50$). $SR_{fit,e}$: success rate from fit on experimental data, $SR_{fit,s}$: success rate from fit on simulated data.

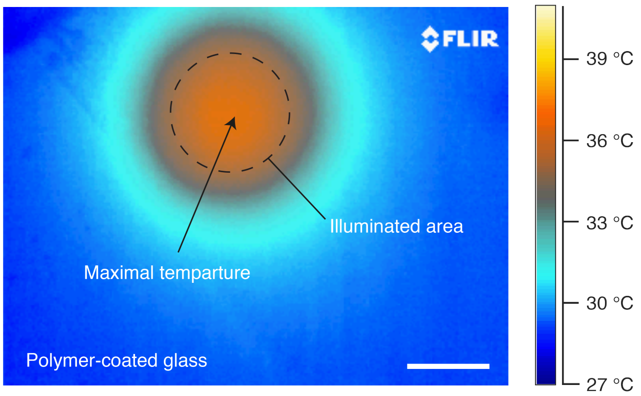

Supplementary Figure 7 — Heat map of P3HT:PCBM during illumination. We quantified the temperature increase upon illumination with an IR camera. We applied an illumination pulse with the same duration and intensity as in the recording experiment on glass coated with the blend. Data analysis was done with extracted maxima within the illuminated area. Scale bar: 1 mm.

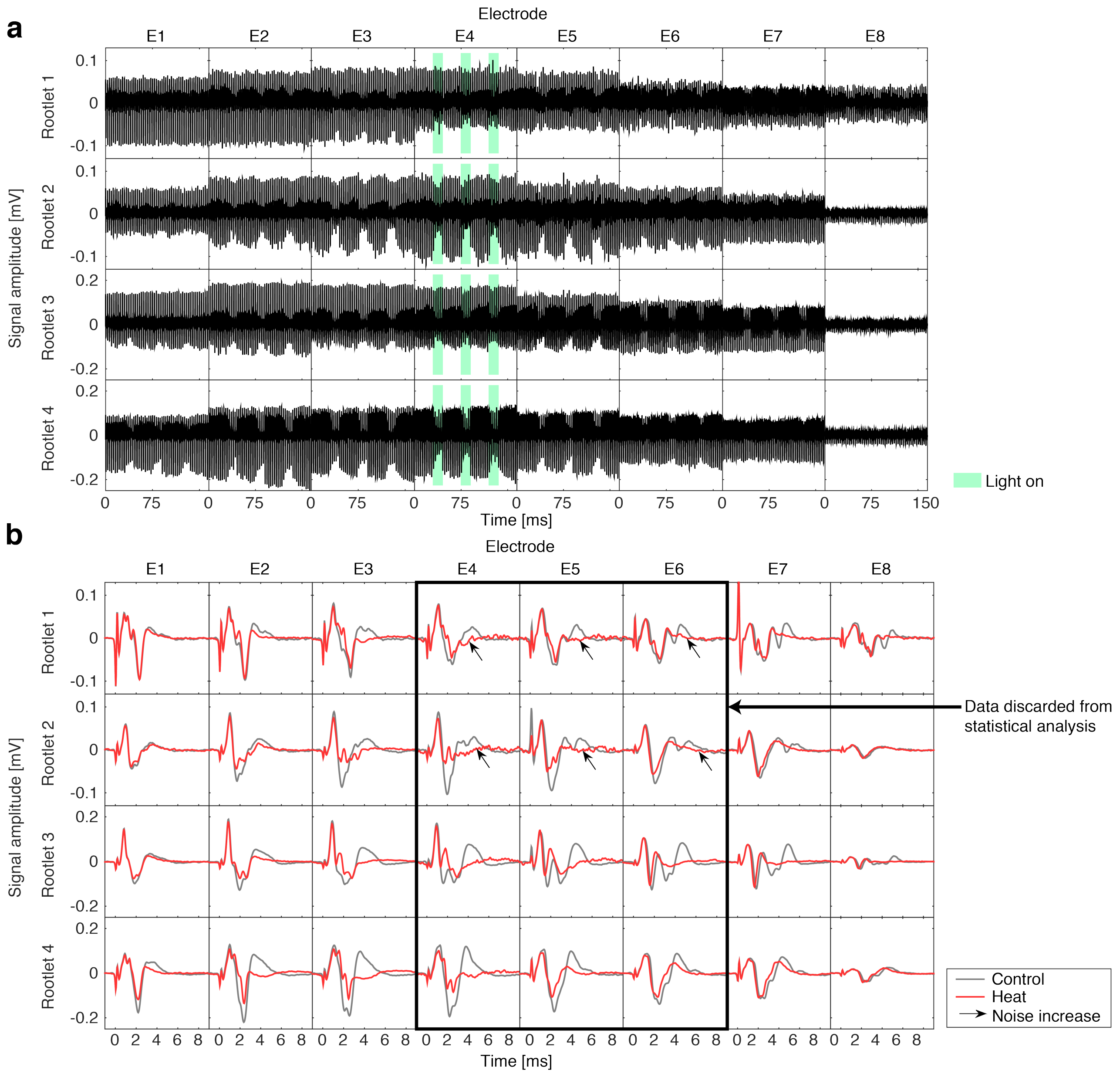

Supplementary Figure 8 — Signal envelope and recordings of all four rootlets. a, Concatenated raw data for all recordings. Nerve signals after each electrical stimulation were isolated in a 8 ms window and concatenated. Windows started 1 ms after the electrical stimulation to remove stimulation artifacts. Light pulse were illuminating electrode E4. **b,** Averaged data for all recordings. MUAP obtained at the end of the heating phase (Heat) and the cooling phase (Control) were averaged across light pulses (applied 3 times per rootlet). MUAP initially propagated as a single wave (E1) and spread out downstream into 2-3 spikes. On electrode E7, we can observe a stronger inhibition of spikes that have a propagation delay > 4 ms. We used this delay to distinguish between slow and fast fibers (see Supplementary Fig. 9). The heat increased the white noise on ITO electrodes (electrode E4, E5 and E6, see arrows), and therefore these recordings were discarded from the data analysis.

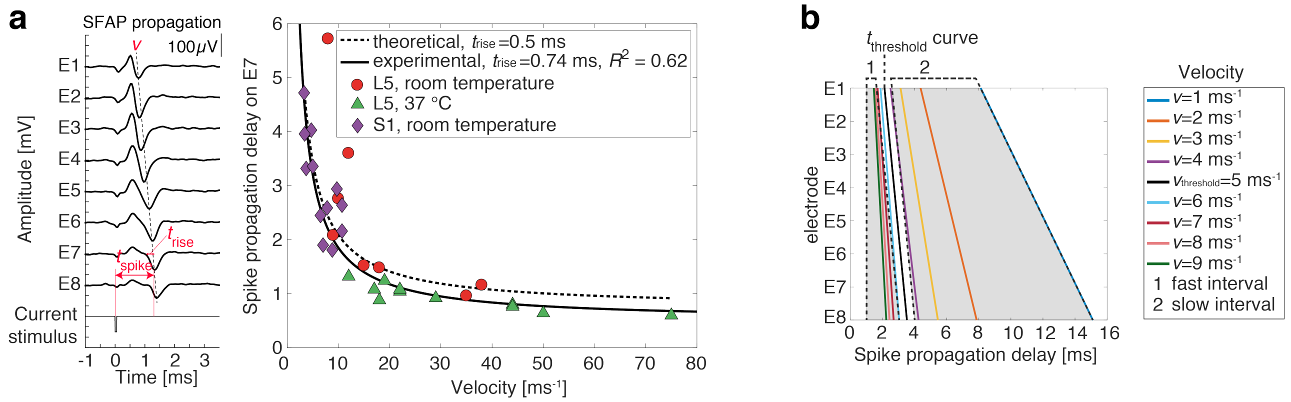

Supplementary Figure 9 — Calculation of NSD integration interval for fast and slow fibers. a, Experimental and theoretical relation between spike delay and velocity. Nerve signal from ventral L5 rootlet were elicited at room temperature and at 37°C. Nerve signal of rootlet from dorsal S1 were elicited at room temperature. Spike delay at E7 were measured in SFAP and MUAP and plotted against their corresponding velocities using Supplementary Equation 14 (with d = 13.5 mm). Experimental data fit lead to t_{rise} = 0.74 ms, therefore validating our approximation. R^2: R-squared of the fit. **b,** Integration interval of slow and fast fibers in function of the electrode position. We used Supplementary Equation 14 to calculate propagation delay at each electrode for velocities between 1 and 9 ms^{-1}. The values of $t_{\text{threshold}}$ correspond to a velocity of 5 ms^{-1}. Since a SFAP has an average duration of 1 ms, the band around $t_{\text{threshold}}$ was removed in order to avoid analyzing SFAPs that could be overlapping the slow and fast NSD; integration of fast fiber started 1 ms after the stimulation to remove the channel entry artefact and stopped 0.5 ms before $t_{\text{threshold}}$. Integration of slow fibers started 0.5 ms after $t_{\text{threshold}}$ and stopped at the end of the MUAP, when down to 2 μV.

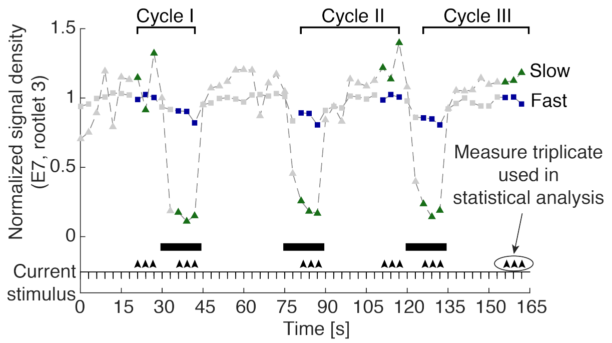

Supplementary Figure 10 — Statistical parameters. a, Illustration of statistical parameters on the NSD of slow and fast fiber summarized in Supplementary Table 3. Kinetics of inhibition was not taken into account in this statistical analysis. Instead, measure triplicates corresponding to stabilized value of the NSD (after a temperature change), highlighted by arrows, were used in this analysis.

Effect influencing the inhibition	Factor name	Factor levels (number of levels)
Distance of the heating source	*electrode*	E1, E2, E3, E7, E8 (5)
Temperature change	*illumination*	light on, light off (2)
Diameter of fibers	*fiber type*	slow, fast (2)
Reversibility of the inhibition	*treatment repetition*	heating/cooling cycle I, II, III (3)

Supplementary Table 3 — Statistical parameters Summary of statistical parameters illustrated in a. The experimental design was factorial as the signal density was measured for each combination of factors level.

Statistics	Factor name	*p*-value
Four-way ANOVA: main effect and interaction	*illumination*	0.000329
	fiber type	0.00521
	fiber type x illumination	0.00337
	electrode	0.170
	treatment repetition	0.983
	illumination x treatment repetition	0.221
Post-hoc one-way ANOVA: fast fiber	*illumination*	0.202<br>adjusted with Bonferroni
Post-hoc 1one-way ANOVA: fast fiber	*illumination*	0.0107<br>adjusted with Bonferroni

Supplementary Table 4 — *p*-values of factors main and interaction effect from mixed and *post-hoc* ANOVA. Details on the statistical test can be found in Supplementary Methods 6. Interactions between two factors are indicated by "*x*". As we perform two *post-hoc* tests, we divided the *p*-values by two in order to apply Bonferroni correction. Final *p*-values are shown.

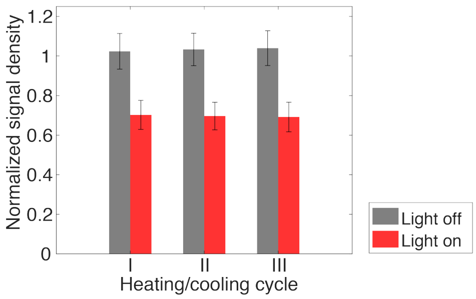

Supplementary Figure 11 — Calculation of NSD and statistical analysis.
Average of the four rootlets across the factors "fiber type" (2x) and "electrode" (5x) There was no significant effect of heating/cooling cycle repetitions ($p = 0.983$, indicated in Supplementary Table 4) and no significant interaction between heating/cooling cycle and illumination ($p = 0.221$, indicated in Supplementary Table 4), suggesting that the inhibition effect was reversible and repeatable. Error: pooled standard error (n = 4, each repeated 10x).

Supplementary Methods

Building a VSR performance model using preliminary SFAP simulation

Modeling and statistics were done in MATLAB 2016b. In this study, we were interested in understanding how fabrication parameters would influence the performance of the VSR algorithm and which SFAP velocities could be measured accurately as a function of their waveform characteristics. SFAP were simulated using a trans-membrane action potential function[2]:

$$V_{\mathrm{m}}(t) = -At^{n}e^{-Bt} \quad \textbf{(1)}$$

where A and B determine the SFAP amplitude and width at half prominence, and n the SFAP waveform (here we used $n = 1$ and obtained monophasic spikes). Donaldson group defined the velocity quality factor (Q_v) to assess the performance of the VSR algorithm.[2] It is the ratio between the propagation velocity, v, and the intrinsic velocity spectrum (IVS) width at a fixed attenuation (Supplementary Fig. 4a):

$$Q_v = \frac{v}{v_{+} - v_{-}} \quad \textbf{(2)}$$

where, v_{+} and v_{-} are the velocities at which max(IVS) is attenuated of $\alpha = 0.1$ dB. Q_v was measured in function of SFAP amplitude, width and velocity as well as electrode number and pitch using simulated data (without added noise). Q_v is a linear function of $e^{\frac{1}{v}}$, width, N_{E} and pitch and does not depend on SFAP amplitude ($R^2 = 0.93$ - 1.00, Supplementary Fig. 4b). To obtain the Q_v model, all linear terms were summed up into a single equation:

$$\text{Performance} \sim Q_v = a_0 + a_1 \cdot N_{\mathrm{E}} + a_2 \cdot \text{pitch} + a_3 \cdot \text{width} + a_4 \cdot e^{\frac{1}{v}} \quad \textbf{(3)}$$

where a_{i} are the model coefficients. The SFAP amplitude does not affect Q_v and consequently, neither the SNR. However, the SNR introduces the probability of an error of calculation. Thus, we next used the success rate (SR) to quantify the performance. We hypothesized that it is affected by all parameters similarly as Q_v, and dependent on a function f(SNR) leading to the following model:

$$\text{Performance} \sim \text{SR} = a_0 + a_1 \cdot N_{\mathrm{E}} + a_2 \cdot \text{pitch} + a_3 \cdot \text{width} + a_4 \cdot e^{\frac{1}{v}} + a_5 \cdot \text{f(SNR)} \quad \textbf{(4)}$$

In order to find the SNR term and complete the model, we added white Gaussian noise to the simulation, mimicking on SNR and amplitude amplification along the channel (10 mm microchannel, 8 electrodes, 1 mm pitch). The SNR was calculated at the position of maximal amplification (electrode E6, Supplementary Fig. 2, Supplementary Table 1) using[3]:

$$\text{SNR} = 10 \cdot \log_{10}\left(\frac{\text{SFAP}_{\text{amplitude}}}{\text{rms}_{\text{noise}}}\right)^2 \quad \textbf{(5)}$$

where the $\text{rms}_{\text{noise}}$ is the root mean square of the noise. Data filtering and interpolation were done as follows:

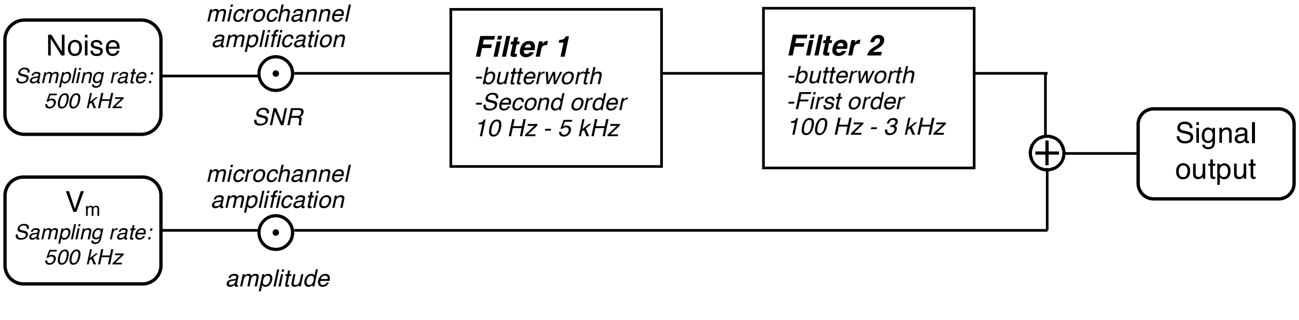

Each SFAP (with varying SNR and velocity) was simulated 10 times to enable the calculation of the SR. For each simulated SNR, we varied the velocity in order to investigate potential interaction effect (between the SNR and the velocity). We repeated the simulation in order to calculated the SR average of 10 trials. The resulting relationship between SR and the SNR is a sigmoid function, dependent on the velocity

(Supplementary Fig. 4c). However, the slope of the sigmoid is independent of the velocity. Thus, we expressed SR as a linear function of the SNR between 0 and 1, thereby removing the SNR/velocity interaction. The final VSR performance model lead to:

$$\text{Performance} \sim \text{SR} = \begin{cases} 1 & \text{if } SR > 1 \\ a_0 + a_1 \cdot N_\text{E} + a_2 \cdot \text{pitch} + a_3 \cdot \text{width} + a_4 \cdot e^{\frac{1}{v}} + a_5 \cdot \text{SNR} & \text{if } 0 < SR < 1 \\ 0 & \text{if } SR > 1 \end{cases} \quad \textbf{(6)}$$

Simulation of SFAP recording in microchannels to fit the VSR performance model

As previously, we simulated SFAP with added Gaussian white noise. Following biological range in Supplementary Fig. 3a, we chose the following parameters values:

$$\text{amplitude} = 50\ \mu\text{V}$$
$$\text{width} \in [0.15, 0.20, \dots, 0.60]\ \text{ms}$$
$$\text{velocity} \in [5, 10, \dots, 60]\ \text{ms}^{-1}$$
$$\text{SNR} \in [0, 2.5, \dots, 50]\ \text{dB}$$

For each platform design (see Supplementary Table 1), we simulated SFAP with all possible combination of width, SNR and velocity. Amplification along the channel and biological ranges were included according to the simulated number of electrode and channel length.

Model fitting and statistics

The model was fitted on the data, and model coefficients a_i were calculated using the least squares fit resolution using the following equations[4]:

$$Y = X\vec{\boldsymbol{a}} + \vec{\boldsymbol{\epsilon}} \Leftrightarrow \begin{pmatrix} SR_1 \\ \vdots \\ SR_n \end{pmatrix} = \begin{pmatrix} 1 & N_{\text{E}_1} & \text{pitch}_1 & \text{width}_1 & e^{\frac{1}{v_1}} & \text{SNR}_1 \\ \vdots & \vdots & \vdots & \vdots & \vdots & \vdots \\ 1 & N_{\text{E}_p} & \text{pitch}_p & \text{width}_n & e^{\frac{1}{v_p}} & \text{SNR}_p \end{pmatrix}_{m\,\text{x}\,p} \begin{pmatrix} a_0 \\ \vdots \\ a_5 \end{pmatrix} + \begin{pmatrix} \epsilon_1 \\ \vdots \\ \epsilon_p \end{pmatrix} \quad \textbf{(7)}$$

$$\text{least squares fit} \Rightarrow \vec{\boldsymbol{a}} = (X^TX)^{-1}X^T\text{Y} \quad \textbf{(8)}$$

where Y is the *response vector*, X is the *model matrix* of dimension m x p, $\vec{\boldsymbol{a}}$ is the *model coefficients* and $\vec{\boldsymbol{\epsilon}}$ is the *residue*. We performed a 5-way ANOVA (two-tail, ss type III, all five parameters were defined as continuous) to test the significance of each term of the linear model. The confidence interval were calculated as follows:

$$CI_{95\%} = t_{\frac{\alpha}{2},df}\sqrt{D_\text{ii}s^2} \quad \textbf{(9)}$$

where D_ii are the diagonal element of the *dispersion matrix*:

$$D = (X^TX)^{-1} \quad \textbf{(10)}$$

s^2 is the *coefficient variance*:

$$s^2 = \frac{\vec{\boldsymbol{\epsilon}}^T\vec{\boldsymbol{\epsilon}}}{df} \quad \textbf{(11)}$$

where df is the *degree of freedom* of the fitting:

$$df = m - n \quad \textbf{(12)}$$

and $t_{\frac{\alpha}{2},df}$ is the two-tail Student Quantile. We used $\alpha = 0.05$ as threshold for significance. For experimental

fit, $df = 208$ and $t_{\frac{\alpha}{2},df} = 1.97$ and for simulation fit, $df = 10'456$ and $t_{\frac{\alpha}{2},df} = 1.97$. In order to compare the relative sensitivity of SR to each terms of the model, we expressed the coefficients a_i as relative half-effect; we normalized each parameter's range of values on the interval [-1.1] prior applying the least square fit (e.g. the range of values for the number of electrodes, $N_E \in [3, 4, 5, 6, 7, 8]$, became $N_{E,std} \in [1, -0.6, -0.2, 0.2, 0.6, 1]$ and were replaced accordingly in the *model matrix*).

Heating of P3HT:PCBM during illumination

We quantified the heating upon illumination with an IR camera (Supplementary Fig. 7, Supplementary Movie 1). We applied an illumination pulse with the same duration and intensity as in the recording experiment on plain glass and glass coated with the polymer. We plotted the maximal temperature over time $T(t)$ and fitted the data with the following equation:

$$T(t) = T_{\text{final}} + (T_{\text{initial}} - T_{\text{final}}) \cdot \left(1 - e^{\frac{t}{\tau_{\text{polymer}}}}\right) \quad \textbf{(13)}$$

where τ_{polymer} is the time constant for the heating of the polymer, T_{initial} is the initial polymer temperature and T_{final} is the final temperature. The R-squared of the fit, R^2, was calculated for the fit.

Calculation of NSD integration interval for fast and slow fibers

In order to distinguish larger fiber activity from thinner fibers activity, we used the velocity as a surrogate variable for fiber diameter. Elicited nerve signals spread into two signal onsets at electrode E7. The time delay separating the two onsets $t_{\text{threshold}}$ (Supplementary Fig. 9a) was chosen to classify SFAP into two fiber type categories: fast (> 5 ms^{-1}) and slow (< 5 ms^{-1}). Spike propagation velocity can be calculated from propagation delay using the following equation:

$$t_{\text{spike}}(v) = t_{\text{rise}} + \frac{d}{v} \quad \textbf{(14)}$$

All variables are illustrated in Supplementary Fig. 9a, where t_{spike} is the time at which the SFAP maximum is detected, t_{rise} is the rising delay between SFAP onset and SFAP maximum, d is the distance between the recording electrode and the stimulation electrodes and v is the SFAP velocity (Supplementary Fig. 9). We approximated t_{rise} as half-duration of a SFAP (in average: $t_{\text{rise}} = 0.5$ ms) and verified its value experimentally. Absolute values of slow and fast neural signal were integrated over the corresponding interval (shown in Supplementary Fig. 9b).

Statistical model to investigate the inhibition mechanisms

We used R-studio software to do the statistical analysis. We measured the NSD inhibition by heat and investigated what modulates the intensity of the inhibition to better understand the effect of the polymer. We were interested in how the distance from the heating source (electrode position with respect to heat source) and the fiber size (nerve response velocity) modulates the inhibition and in knowing if the inhibition was reversible (alternation between heating and cooling the nerve rootlet). Our statistical analysis evaluate the effect of four *factors* on the response variable: NSD, as well as their interaction (e.g., does the effect of the heat varies with the fiber size?)

To do so, we accounted for the effect of 4 factors on the NSD (summarized in Supplementary Fig. 10 and Supplementary Table 3), namely: (i) *electrode* (5 levels: E1, E2, E3, E7, E8), (ii) *illumination* (2 levels: light off, light on), (iii) *fiber type* (2 levels: fast, slow), and (iv) *treatment repetition* (3 levels: cooling/heating I, cooling/heating II, cooling/heating III). The NSD measurement was repeated three times (factor: measure triplicates) for each combination of *electrode*, *illumination*, *fiber type* and *treatment repetition*.

The data follows a symmetric distribution and does not violate the requirement of sphericity. Thus, we were able to use a four-way mixed ANOVA (two-tail, ss type III, all four factors were defined as categorical). The *fiber type* was considered as a *between-subject factor* as it represents two distinct axon populations (fast and slow). All other factors were measured within each axon population, thus computed as a *within-subject factor* (repeated measurements). We tested *factors main effect* and *interaction of pairs of factors* (mix of two main effects). An interaction was considered significant only when the two main effects and the interaction

were significant. (We used $\alpha = 0.05$ for statistical significance). Resulting p-values are shown in Supplementary Table 4.

The main effect *illumination* effect was significant, suggesting the inhibition of the NSD by heat. However, the interaction *illumination* x *fiber type* and the main effect *fiber type* were as well significant, demonstrating that the NSD inhibition is different for slow and fast fibers. We next used *post-hoc* tests to measure the *illumination* effect on the NSD of each type of fiber separately. One-way ANOVA (two-tail, ss type III, factor: *illumination*) was used, first, on data from slow fibers, and second, on data from fast fibers. Bonferroni correction was applied to counteract the problem of multiple comparisons (all p-values were divided by the number of *post-hoc* tests: 2). Resulting p-values are shown in Supplementary Table 4. (We used $\alpha = 0.05$ for statistical significance).

The main effect of the *treatment repetition* and the interaction: *treatment repetition* x *illumination* were not significant demonstrating the reversibility of the inhibition when light is turned off (Supplementary Fig. 11).

The main effect *electrode* is not significant although we observe a trend in the data (Fig. 3g). One the reason could be that the electrode position is not linearly correlated to a change in NSD. Replacing the electrode position by the corresponding distance to the heating source could lead to a better statistical model but is out of the scope of this study.

Inhibition kinetics

Nerve signal density of slow and fast fiber across all the data (except from electrode E4, E5 and E6) were averaged and fitted with the following equation:

$$\mathrm{NSD}_{\mathrm{fast}}(t) = \mathrm{NSD}_{\mathrm{final,fast}} + \left(\mathrm{NSD}_{\mathrm{initial,fast}} - \mathrm{NSD}_{\mathrm{final,fast}}\right) \cdot \left(1 - e^{\frac{t}{\tau_{\mathrm{fast}}}}\right) \quad \textbf{(15)}$$

$$\mathrm{NSD}_{\mathrm{slow}}(t) = \mathrm{NSD}_{\mathrm{final,slow}} + \left(\mathrm{NSD}_{\mathrm{initial,slow}} - \mathrm{NSD}_{\mathrm{final,slow}}\right) \cdot \left(1 - e^{\frac{t}{\tau_{\mathrm{slow}}}}\right) \quad \textbf{(16)}$$

where $\tau_{\mathrm{fibertype}}$ is the time constant for the inhibition of the signal density, $\mathrm{NSD}_{\mathrm{initial,fibertype}}$ is the initial signal density (always equal to 1), and $\mathrm{NSD}_{\mathrm{final,fibertype}}$ is the final signal density. The R-squared of fit, R^2, was calculated for both fits.

Supplementary Note

Amplification along microchannel

Narrow conduits artificially increase the electrical resistance of the extracellular medium thereby proportionally increases the amplitude of the extracellular signals.[1,5] During an action potential, the current i flows across the membrane at the nodes of Ranvier, inducing a change in electrical potential between two nodes of Ranvier. The extracellular difference of potential between two nodes of Ranvier is δV_{out} while the intracellular one is δV_{in}, defined by:

$$\delta V_{\mathrm{out}} = iR_{\mathrm{ecf}} \quad (17)$$

$$\delta V_{\mathrm{in}} = -iR_{\mathrm{axon}} \quad (18)$$

where R_{ecf} is the extracellular fluid resistance and R_{axon} is the axonal resistance. By combining the two equations, we obtain:

$$\frac{\delta V_{\mathrm{out}}}{\delta V_{\mathrm{in}}} = -\frac{R_{\mathrm{ecf}}}{R_{\mathrm{axon}}} \quad \text{with } R_{\mathrm{axon}} \gg R_{\mathrm{ecf}} \quad (19)$$

which illustrate how increasing the extracellular fluid resistance increases the voltage difference measured between the two nodes of Ranvier. The resistance depends on the dimension of the conductive volume as follows:

$$R_{\mathrm{ecf}} = \frac{l_{\mathrm{microchannel}} \cdot \rho_{\mathrm{ecf}}}{S_{\mathrm{microchannel}}} \quad (20)$$

where R_{ecf} is the extracellular fluid resistance inside the microchannel, $l_{\mathrm{microchannel}}$ is the length of the microchannel (of the conductive volume), ρ_{ecf} is extracellular fluid resistivity and $S_{\mathrm{microchannel}}$ is the cross-sectional area of the microchannel (or of the conductive volume).

Therefore, restricting the conducting volume using a microchannel increases the extracellular fluid resistance, which in turns increases the measured potential. Moreover, Supplementary Equations 19 and 20 show that longer and/or narrower microchannel will increase the amplification of nerve fiber signals.

Supplementary References

[1] FitzGerald, J. J., Lacour, S. P., McMahon, S. B., & Fawcett, J. W. (2009). Microchannel Electrodes for Recording and Stimulation: In Vitro Evaluation. *IEEE Transactions on Biomedical Engineering*, *56*(5), 1524–1534.

[2] Martin Schuettler, Nick Donaldson, Vipin Seetohul, and John Taylor. Fibre-selective recording from the peripheral nerves of frogs using a multi-electrode cuff. *Journal of Neural Engineering*, 10(3):036016, June 2013.

[3] Joon Hwan Choi, Hae Kyung Jung, and Taejeong Kim. A new action potential detector using the MTEO and its effects on spike sorting systems at low signal-to-noise ratios. *IEEE Transactions on Biomedical Engineering*, 53(4):738–746, April 2006.

[4] Douglas C Montgomery. Design and Analysis of Experiments. *John Wiley & Sons*, 7th edition, July 2008.

[5] FitzGerald, J. J., Lacour, S. P., McMahon, S. B., & Fawcett, J. W. (2008). Microchannels as Axonal Amplifiers. *IEEE Transactions on Biomedical Engineering*, *55*(3), 1136–1146. http://doi.org/10.1109/TBME.2007.909533
