# Supplementary material for: A microfabricated nerve-on-a-chip platform for rapid assessment of neural conduction in explanted peripheral nerve fibers
Source: Nat Commun. 2018 Oct 23;9:4403. doi: 10.1038/s41467-018-06895-7 (PMC6199302; doi:10.1038/s41467-018-06895-7)
Supplement: Supplementary file 2 — Description of Additional Supplementary Files [file 41467_2018_6895_MOESM2_ESM.pdf]

Description of Additional Supplementary Files

File Name: Supplementary Movie 1
Description: Rootlet dissection and experimental setup.

File Name: Supplementary Movie 2
Description: MUAP recording played over time.

File Name: Supplementary Movie 3
Description: Heat map over time of P3HT:PCBM during and after illumination.
